# Genetic Features of Metachronous Esophageal Cancer Developed in Hodgkin’s Lymphoma or Breast Cancer Long-Term Survivors: An Exploratory Study

**DOI:** 10.1371/journal.pone.0117070

**Published:** 2015-01-22

**Authors:** Elisa Boldrin, Enrica Rumiato, Matteo Fassan, Rocco Cappellesso, Massimo Rugge, Vanna Chiarion-Sileni, Alberto Ruol, Rita Alfieri, Matteo Cagol, Carlo Castoro, Alberto Amadori, Daniela Saggioro

**Affiliations:** 1 Immunology and Molecular Oncology, Veneto Institute of Oncology, IOV-IRCCS, Padova, Italy; 2 Department of Medicine, Surgical Pathology and Cytopathology, University of Padova, Padova, Italy; 3 Medical Oncology, Veneto Institute of Oncology, IOV-IRCCS, Padova, Italy; 4 Department of Surgical Sciences, Oncology and Gastroenterology, University of Padova, Padova, Italy; 5 Oncological Surgery, Veneto Institute of Oncology, IOV-IRCCS, Padova, Italy; Institut Jacques Monod, FRANCE

## Abstract

**Background:**

Development of novel therapeutic drugs and regimens for cancer treatment has led to improvements in patient long-term survival. This success has, however, been accompanied by the increased occurrence of second primary cancers. Indeed, patients who received regional radiotherapy for Hodgkin’s Lymphoma (HL) or breast cancer may develop, many years later, a solid metachronous tumor in the irradiated field. Despite extensive epidemiological studies, little information is available on the genetic changes involved in the pathogenesis of these solid therapy-related neoplasms.

**Methods:**

Using microsatellite markers located in 7 chromosomal regions frequently deleted in sporadic esophageal cancer, we investigated loss of heterozygosity (LOH) and microsatellite instability (MSI) in 46 paired (normal and tumor) samples. Twenty samples were of esophageal carcinoma developed in HL or breast cancer long-term survivors: 14 squamous cell carcinomas (ESCC) and 6 adenocarcinomas (EADC), while 26 samples, used as control, were of sporadic esophageal cancer (15 ESCC and 11 EADC).

**Results:**

We found that, though the overall LOH frequency at the studied chromosomal regions was similar among metachronous and sporadic tumors, the latter exhibited a statistically different higher LOH frequency at 17q21.31 (p = 0.018). By stratifying for tumor histotype we observed that LOH at 3p24.1, 5q11.2 and 9p21.3 were more frequent in ESCC than in EADC suggesting a different role of the genetic determinants located nearby these regions in the development of the two esophageal cancer histotypes.

**Conclusions:**

Altogether, our results strengthen the genetic diversity among ESCC and EADC whether they occurred spontaneously or after therapeutic treatments. The presence of histotype-specific alterations in esophageal carcinoma arisen in HL or breast cancer long-term survivors suggests that their transformation process, though the putative different etiological origin, may retrace sporadic ESCC and EADC carcinogenesis.

## Introduction

It is well established that therapeutic irradiation can favor the development of a second primary malignancy several years later. Indeed, given the improvement of anti-cancer therapeutic regimens, the occurrence of therapy-related metachronous tumors is rising along with the increased survival. Notably, long-term survivors previously treated for Hodgkin’s Lymphoma (HL) or breast cancer may develop a solid metachronous tumor in the irradiated area [1, 2]. Among the treatment-related metachronous tumors, esophageal carcinoma has also been reported [3, 4] and previous studies have documented an increased esophageal cancer risk in breast cancer long-term survivors, possibly related to radiotherapy [5, 6].

The two predominant sporadic esophageal cancer histotypes are the squamous cell carcinoma (ESCC) and the adenocarcinoma (EADC) that are considered to have different etiological origin. The risk factors for ESCC are believed to be smoking, alcohol abuse and chronic inflammation, whereas EADC is mainly associated with gastro-esophageal reflux and obesity [7–9]. These two histologies are also represented in second primary esophageal carcinoma.

Although several studies have investigated the epidemiology of therapy-related metachronous tumors, only little information on their genetic abnormalities is available and there are no data on therapy-related esophageal cancers. Esophageal carcinomas arise following a multistep process and the acquisition of genetic alterations is tightly related to tumor progression.

Loss of heterozygosity (LOH) studies, conventional comparative genomic hybridization (CGH) and multiplex ligation-dependent probe amplification (MLPA) analyses demonstrated the genetic complexity behind the esophageal carcinogenesis. ESCC and EADC are characterized by recurrent losses or gains, some common and others unique, at chromosomal regions 3p, 4q, 5p, 5q, 8p, 9p, 13q, and 21q [10–14]. In particular, deletions at chromosomal region 3p26.3-p14.2, that contains candidate tumor suppressor genes as *FHIT*, *TGFBR2*, *FBLN2* and *WNT7A*, are one of the most frequent abnormalities in both ESCC and EADC [15–17], as well as deletions at 5p12, 5q11.2-q14.3 and 5q31 [11–13, 18]. Using MLPA analyses, we previously reported that the region containing *MSRA* and *CTSB* (8p23.1) genes is lost at high frequency in ESCC [12]. A very close region (8p23.2-p21.3), containing the candidate tumor suppressor genes *DLC1*, *LZTS1*, *MTUS1* and *PSD3*, was found to be an LOH hot-spot in ESCC by other groups [11, 15, 19]. Conversely, chromosome 4 exhibits high LOH frequency in EADC, which, however, is characterized by a more widespread genomic instability [12, 18]. Several studies have shown the frequent allelic loss at 9p21.3 chromosomal band, containing the tumor suppressor genes *CDKN2A* and *CDKN2B*, suggesting that their inactivation could be relevant for ESCC and EADC development [15, 18–21]. In ESCC, and less frequently in EADC, LOH was also reported for the region 13q13.1-q14.2 containing the tumor suppressor genes *BRCA2* and *RB* [10, 12, 15, 22], and for the 17q21.3 that includes the tumor suppressor gene *BRCA1* [16, 23, 24]. Conflicting results concerning the presence of microsatellite instability (MSI) in esophageal cancer are available, with the majority of reports showing low MSI frequency in both ESCC and EADC [25–28].

As mentioned before, only few studies investigated the genetic alterations of therapy-related second primary cancers, and they mainly concern lung or breast cancer developed in HL long-term survivors [29, 30]; so far, no data are available on therapy-related metachronous esophageal carcinomas, probably due to their rather low incidence.

In this study, for the first time, we analyzed at the molecular level esophageal cancers developed in HL or breast cancer long-term survivors to address the question whether they are characterized by peculiar chromosomal changes that distinguish them from the sporadic counterpart.

## Materials and Methods

### Selection of patients and sample collection

Formalin fixed paraffin-embedded (FFPE) surgical specimens from tumor and matched normal tissues (i.e. distant from the tumor) of 20 patients with metachronous esophageal cancer, collected from 1991 to 2010, were analyzed. Among them 14 were ESCC and 6 EADC, 12 developed in HL and 8 in breast cancer long-term survivors. As a cure for the first neoplasia, all patients received gamma radiation (range 36–40 Gy for HL and 50 Gy for breast cancer) together with chemotherapeutic drugs, mainly alkylating and/or antimitotic agents. The latter, contrary to gamma radiation, have changed in both typology and dosage during the long period of sample collection. As controls, 26 paired FFPE surgical specimens of sporadic esophageal cancers (15 ESCC and 11 EADC), selected from consecutive patients diagnosed within 2004–2008, were included in the study. All the cases considered were retrieved and reviewed from the archives of the Surgical Pathology and Cytopathology Unit at the University of Padova. Samples were obtained from patients who underwent esophagectomy without pre-operative radiotherapy or chemotherapy. For some patients we also collected peripheral blood lymphocytes, and the extracted DNA was used as internal control to assess the reproducibility of data obtained from the FFPE-derived DNA. The study had the consent of the Research Ethics Committee of the Veneto Institute of Oncology, IOV-IRCCS, Padova, Italy. The Research Ethics Committee waived informed consent of patients due the retrospective nature of the study. Prior to analysis, a numerical code was assigned to each sample in order to anonymize and de-identify them.

DNA was extracted from 7–10 × 10 μm sections of FFPE samples using the QIAamp DNA Mini kit following the manufacturer’s instruction (Qiagen, Milano, Italy) or an automated DNA extractor (MagNA Pure Compact, Roche, Milano, Italy). The concentration and quality of the extracted DNA were tested with NanoDrop 1000 spectrophotometer (Agilent Technologies, Santa Clara, CA, USA); no difference in DNA quality was observed between the two methods used. Tumor samples exhibiting a neoplastic cellularity lower than 70% were enriched in the neoplastic component by manual macrodissection using a hematoxylin and eosin stained slide as a guide for the unstained serial slides. DNA from peripheral blood samples was extracted with the Flexigene kit (Qiagen, Milano, Italy).

### LOH and MSI analyses

DNAs from tumor and matched normal samples were analyzed using primers for the following microsatellite markers: D3S3727 (3p24.1), D5S2106 (5p12), D5S623 (5q11.2), D8S1130 (8p23.1), D9S942 and D9S171 (9p21.3), D13S260 (13q13.1), D13S267 (13q13.2), D17S1323 and D17S1327 (17q21.31); the forward primers were labeled at 5’-end with FAM, HEX or VIC fluorescent dyes (Sigma Aldrich, Milano, Italy). DNA was amplified in a 10 μl total polymerase chain reaction (PCR) volume containing PCR Gold buffer (Life technologies, Monza, Italy), 250 μM each of dATP, dCTP, dGTP, dTTP (Life technologies, Monza, Italy), 0.4 μM each of forward and reverse oligonucleotide primers, and 1 μl of Q-solution (Qiagen, Milano, Italy). A DNA amount of 20–30 ng was used in each PCR, and, depending on the PCR reaction, we utilized MgCl_2_ and AmpliTaq Gold DNA polymerase (Life technologies, Monza, Italy) at concentrations ranging from 1.5 to 2.5 mM and 0.04 to 0.1 Units, respectively; details are reported in the supplementary S1 Table. PCR reaction product was analyzed by capillary electrophoresis using the 3730xl DNA analyzer (Life technologies, Monza, Italy).

LOH was defined as a reduction of ≥30% in the fluorescent signal of one allele in the tumor sample compared with its normal counterpart. Samples were considered MSI positive if new peaks or changes in the microsatellite fragments were present in the tumor sample compared with its normal counterpart. The fractional allelic loss (FAL) index was calculated for each patient as follows: total number of loci with LOH / total number of informative loci.

### Statistical analysis

Statistical analyses, to assess possible significant differences between therapy-related metachronous esophageal cancer samples and sporadic ones, were performed using the MedCalc software (v.12.2.1). Mann-Whitney and two-tailed Fisher’s exact tests were applied for continuous and categorical variables, respectively. Although this study, due to the low incidence of metachronous esophageal cancers in patients with a past history of HL or breast cancer, should be considered as a pilot study, we calculated that our sample size, setting α at 0.05 and assuming a difference of LOH proportions of 0.45, will reach a power of 80%.

## Results

### Characteristics of patients

A total of 20 metachronous esophageal cancers developed in HL (12 pts) or breast cancer (8 pts) long-term survivors were included in this study; among them 14 were ESCC and 6 EADC arisen after a median latency period of 20 years (range 3–40). Twenty six sporadic esophageal cancers (15 ESCC and 11 EADC) were included as control.

As reported in Table 1, the clinico-pathological characteristics of both groups were similar. However, given the low incidence of primary esophageal cancer in young people, the median onset age in the sporadic esophageal cancer group was higher than that observed in the treatment-related metachronous tumors (62 years, range 39–83 vs. 53 years, range 26–77). Women were over-represented in the metachronous cancer group (75% vs. 23%) because a consistent part of these tumors developed in female breast cancer long-term survivors. However, a high prevalence of second primary esophageal tumors was also observed in women with a history of past HL (7 out of 12 pts), suggesting a link between the occurrence of a metachronous esophageal carcinoma and the previous therapy. Indeed, sporadic esophageal cancer had usually a rather lower incidence in woman than in man (1:7 ratio) [31, 32].

**Table 1:** Clinico-pathological characteristics of patients with metachronous or sporadic esophageal cancer.

**Characteristics**	**Metachronous**	**Sporadic^a^**	**p-value^b^**
	**Total (n = 20)**	**Total (n = 26)**	
	**n (%)**	**n (%)**	
First primary tumor			
Hodgkin′s Lymphoma	12 (60)	-	/
Breast	8 (40)	-	
Sex			
Male	5 (15)	20 (77)	0.0008
Female	15 (75)	6 (23)	
Histotype			
ESCC	14 (70)	15 (58)	0.54
EADC	6 (30)	11 (42)	
Onset of esophageal cancer (years)			
Median (Range)	53 (26–77)	62 (39–83)	0.03
Latency period (years)			
Median (Range)	20 (3–40)	-	/
pStage			
I	3 (15)	3 (13)	1^c^
II	7 (35)	9 (38)	
III	8 (40)	7 (29)	
IV	2 (10)	5 (20)	

^a^pStage and onset data not available for 2 patients.

^b^p-value was calculated using two-tailed Fisher’s exact test for categorical and Mann-Whitney test for continuous variables.

^c^pStage I–II vs. III–IV.

### Genetic alterations analysis

LOH and MSI frequencies were analyzed using 10 microsatellite markers mapping nearby genes that may contribute to carcinogenesis, and located in 7 chromosomal regions reported as commonly deleted in sporadic ESCC and EADC (i.e. 3p24; 5p12; 5q11; 8p23; 9p21; 13q13; 17q21) (Table 2).

**Table 2:** Microsatellite markers and neighboring genes.

**Microsatellites**	**Ch. location**	**Candidate Gene**	**Distance**	**Gene function^a^**
D3S3727	3p24.1	*TGFBR2*	internal	Cell proliferation
D5S2106	5p12	*DAB2*	5.6 Mb	Mitogen-responsive phospho-protein
		*RAD1*	10 Mb	Cell cycle checkpoint/DSB repair
D5S623	5q11.2	*PDE4D*	6.0 Mb	Member of phosphodiesterase family
D8S1130	8p23.1	*CTSB*	134 Kb	Degradation/turnover of proteins
		*DLC1*	1.1 Mb	Cytoskeletal remodeling
		*MSRA*	2.0 Mb	Repair of oxidative damage
		*MTUS1*	5.6 Mb	Cell proliferation inhibitor
		*PSD3*	6.5 Mb	Guanine nucleotide exchange factor
		*LZTS1*	8.2 Mb	Cell cycle and cell proliferation
D9S942	9p21.3	*CDKN2A*	internal	Cell cycle regulation
		*CDKN2B*	18 Kb	
D9S171	9p21.3	*CDKN2A*	2.51 Mb	Cell cycle regulation
		*CDKN2B*	2.50 Mb	
D13S260	13q13.1	*BRCA2*	352 Kb	DSB/HR repair
D13S267	13q13.2		1.4 Mb	
D17S1323	17q21.31	*BRCA1*	95 Kb	Cell cycle control; HR repair
D17S1327	17q21.31		232 Kb	

^a^According to: www.genecards.org.

At first, given the putative common etiology of the metachronous tumors (i.e. exposure to carcinogens such as gamma radiations and chemotherapeutics), we compared the frequencies of genetic alterations in metachronous ESCC and EADC vs. their sporadic counterpart, to search for specific therapy-related abnormalities, regardless of their histotype. We found that, in therapy-related metachronous esophageal carcinomas the genetic alterations and their frequencies did not significantly differ from those of sporadic cancers with, however, some exceptions: the 8p23.1 and the 17q21.31 regions showing a higher LOH frequency in the sporadic esophageal carcinomas. The difference reached the statistical significance at 17q21.31 (p = 0.018) and only a trend of significance at 8p23.1 (p = 0.07) (Table 3).

**Table 3:** LOH and MSI status detected in metachronous or sporadic ESCC and EADC patients.

**SPORADIC**											
**Patient**	**D3S3727 3p24.1**	**D5S2106 5p12**	**D5S623 5q11.2**	**D8S1130 8p23.1**	**D9S942 9p21.3**	**D9S171 9p21.3**	**D13S260 13q13.1**	**D13S267 13q13.2**	**D17S1323 17q21.31**	**D17S1327 17q21.31**
ESCC										
01	●	○	●	●	○	●	●	Ø MSI	●	●
02	○	○	●	Ø	○	Ø	Ø	●	Ø	○
03	Ø MSI	Ø	○	Ø	○	Ø	●	●	○	●
04	●	Ø	●	Ø	●	Ø	●	Ø	●	Ø
05	●	Ø	Ø	Ø	●	○	Ø	○	Ø	Ø
06	●	○	Ø	○	○	● MSI	Ø	●	○	○
07	●	●	Ø	●	●	●	Ø	●	Ø	●
08	●	Ø	●	Ø	●	●	●	○	●	●
09	●	●	●	●	● MSI	Ø	●	●	●	Ø
10	Ø	●	●	●	○	●	●	●	Ø	○
11	●	○ MSI	○	○	○	○	○	○	○	○
12	○	○	Ø	●	●	●	●	Ø	○	Ø
13	○	●	●	●	○	●	Ø	●	○	○
14	○	○	○	○	○	○	●	Ø	○	○
15	●	Ø	Ø	○	●	●	●	●	○	●
EADC										
01	●	●	●	○ MSI	● MSI	○	○	●	Ø	Ø
02	○	○	Ø	●	○	Ø	Ø	●	Ø	Ø
03	○	○	Ø	●	○	○	Ø	●	Ø	○
04	●	Ø	○	●	○	○	○	●	Ø	●
05	Ø	●	Ø	●	○	●	Ø	●	Ø	●
06	● MSI	Ø MSI	○ MSI	Ø MSI	○	○ MSI	○	○ MSI	○ MSI	●
07	Ø	●	●	Ø	●	●	●	●	●	●
08	○	Ø	○	●	●	Ø	DEL	○	○	●
09	Ø	○	○	Ø	○	●	○	○	Ø	Ø
10	Ø	Ø	○	●	●	●	●	Ø	Ø	Ø
11	●	●	●	●	●	Ø	●	●	●	Ø
Pos/inf (%)	13/20(65)	8/17(47)	10/18(56)	13/18(72)	12/26(46)	12/19(63)	13/18(72)	15/21(71)	6/15(40)	10/17(59)
METACHRONOUS											
Patient	D3S3727 3p24.1	D5S2106 5p12	D5S623 5q11.2	D8S1130 8p23.1	D9S942 9p21.3	D9S171 9p21.3	D13S260 13q13.1	D13S267 13q13	D17S1323 17q21.31	D17S1327 17q21.31
ESCC										
01	●	○	●	●	○	●	Ø	●	Ø	Ø
02	○	Ø	●	Ø MSI	Ø	●	○	●	●	Ø
03	●	●	Ø	Ø	●	○	Ø	●	○	○
04	●	○	Ø	○	○	Ø	Ø	●	○	○
05	○	Ø	●	○	○	Ø	○	Ø	○	○
06	Ø	●	●	Ø	○	●	●	●	○	●
07	●	NA	NA	Ø MSI	●	●	○	NA	NA	Ø
08	●	○	Ø	○	○	●	○	Ø	○	○
09	○	○	●	Ø	○	●	Ø	Ø	Ø	Ø
10	●	○	●	Ø	●	Ø	○	Ø	Ø	○
11	Ø	●	○	●	○	○	●	○	○	○
12	●	●	●	○	●	●	●	Ø	Ø	Ø MSI
13	Ø	●	●	●	●	●	●	●	○	Ø
14	●	○	Ø	○	●	●	Ø	○	Ø	Ø
EADC										
01	○	●	●	○	● MSI	○	●	Ø	Ø	Ø
02	○	Ø	○	○	○	Ø	Ø	○	Ø	○
03	●	●	○	Ø	○	●	Ø	●	Ø	○
04	Ø	Ø	Ø	●	○	●	○	○	●	○
05	Ø	●	○	●	●	○	●	●	Ø	Ø
06	Ø	●	Ø	○	○	○	○	Ø	○	Ø
Pos/inf (%)	9/14(64)	9/15(60)	9/13(69)	5/13(38)	8/19(42)	11/16(69)	6/13(46)	8/12(67)	2/10(20)	1/10(10)
p-value	1	0.50	0.48	0.07	1	1	0.26	1	0.4	0.018

(●) LOH positive; (○) LOH negative; (Ø) uninformative; MSI (positive to microsatellite instability); NA (not amplifiable); DEL: deletion. p-value was calculated using the two-tailed Fisher’s exact test.

When we took into account the histological tumor types, rather than their theoretical etiology, stratifying the metachronous and sporadic cancers by histotype, some degree of genetic specificity came up. Indeed, we found that in both metachronous and spontaneous ESCC the LOH frequencies at chromosomal regions 3p24.1, 5q11.2, 9p21.3 were much higher than in EADC. The difference reached the statistical significance at 5q11.2 region (p = 0.02) and a trend at 9p21.3 region (p = 0.08) (Table 4). No specific alterations at high frequency and shared by metachronous and spontaneous EADC were found; on the contrary, dissimilar frequencies at 5p12 (100% vs. 57%), and 17q21.31 (0% vs. 83%) were observed (Table 4).

**Table 4:** Comparison of LOH frequencies between sporadic and metachronous ESCC and EADC.

**Microsatellites**	**Ch. location**	**ESCC**		**EADC**		**p-value^a^**
		**Sporadic pos/inf (%)**	**Metachronous pos/inf (%)**	**Sporadic pos/inf (%)**	**Metachronous pos/inf (%)**	**ESCC vs EADC**
D3S3727	3p24.1	9/13 (69)	8/11 (73)	4/7 (57)	1/3 (33)	0.43
D5S2106	5p12	4/10(40)	5/11 (45)	4/7 (57)	4/4 (100)	0.15
D5S623	5q11.2	7/10 (70)	8/9 (88)	3/8 (38)	1/4 (25)	**0.02**
D8S1130	8p23.1	6/10 (60)	3/8 (38)	7/8 (88)	2/5 (40)	0.46
D9S942	9p21.3	7/15 (47)	6/13 (46)	5/11 (45)	2/6 (33)	0.77
D9S171	9p21.3	8/11 (73)	9/11 (82)	4/8 (50)	2/5 (40)	0.08
D13S260	13q13.1	9/10 (90)	4/9 (44)	4/8 (50)	2/4 (50)	0.45
D13S267	13q13.2	8/11 (73)	6/8 (75)	7/10 (70)	2/4 (50)	0.7
D17S1323	17q21.31	4/11 (36)	1/8 (12)	2/4 (50)	1/1 (50)	0.34
D17S1327	17q21.31	5/11 (45)	1/7 (14)	5/6 (83)	0/3 (0)	0.41

^a^ p-value was calculated using the two-tailed Fisher’s exact test.

**Figure 1:**
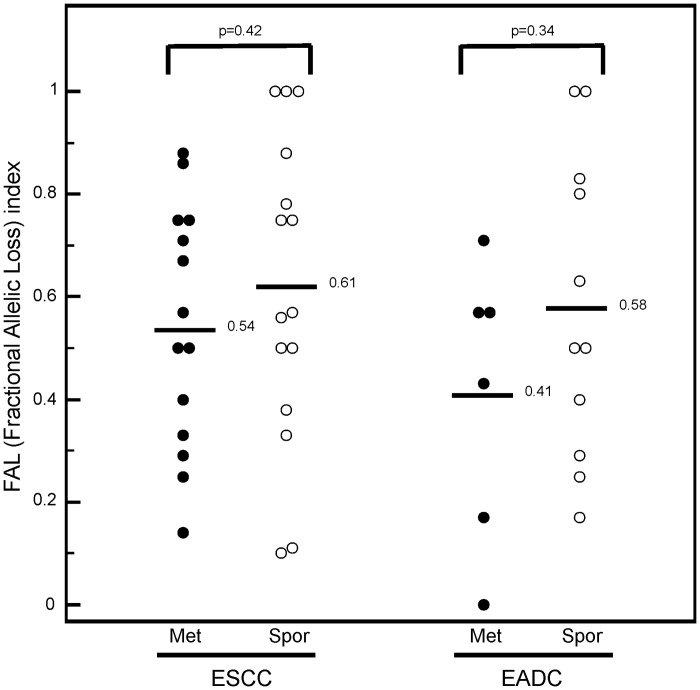
Comparison of LOH in post-HL and post-breast cancer ESCC or EADC and corresponding sporadic form. FAL index is an indicator of LOH at all chromosomal loci analyzed per patient, and it has been calculated as described in Materials and Methods section. P value was calculated using Mann-Whitney test.

Considering the genetic instability at individual level, we observed that both therapy-related metachronous ESCC and EADC had a fractional allelic loss (FAL) index lower than their sporadic counterpart (Fig. 1). MSI analysis showed that metachronous esophageal carcinomas presented the same genomic instability of sporadic tumors. Indeed, by using the same 10 microsatellite markers of the LOH analysis, we observed that at least one MSI event was present in 21% of the therapy-related ESCC patients vs. 33% of the sporadic ones, and in 17% of therapy-related vs. 18% of the sporadic EADC (Table 3).

## Discussion

The high occurrence of metachronous tumors, within or nearby the irradiated area, in HL or breast cancer long-term survivors had suggested a correlation between the development of these malignancies and the previous radiotherapy [1, 2]. Besides the high occurrence of metachronous breast and lung tumors as a consequence of radiotherapy in HL or breast cancer patients, also esophageal cancer has been reported as result of the incidental irradiation [3, 4]. In particular, epidemiological studies have reported an increased incidence of esophageal cancer in women who received radiation for breast cancer [5, 6].

So far, little information is available regarding the genetic alterations that characterize these second primary cancers; in particular, it is not known if they follow specific pathways of carcinogenesis, distinct from those of the sporadic forms. Previous studies reported no significant differences in the pattern of genetic alterations in radiation-induced and spontaneous astrocytomas [33]. By contrast, radiation-associated sarcomas and meningiomas exhibited genetic alterations different from those detected in their sporadic forms [34, 35]. Contradictory results are available concerning second primary lung or breast cancers arisen after therapy for HL, with post-HL lung cancers exhibiting a LOH frequency similar to the sporadic counterpart, and post-HL breast cancers showing a significant higher frequency of allelic loss, compared with sporadic tumors [30]. MSI has also been reported in pediatric second primary neoplasms and post-HL lung and breast cancers [30].

We studied patients with metachronous esophageal cancer developed in long-term survivors who received local regional gamma radiation for a previous HL or breast cancer. The high number of women in post-HL metachronous esophageal cancers (7 out of 12 pts) strongly suggests a causal association between the prior therapy and the second primary tumor onset. More to the point, the incidence of sporadic esophageal tumors in women is much lower.

We are aware that a limitation of the study is the relative low number of analyzed samples mainly due to the fact that therapy-related esophageal cancer is a very rare event. Nonetheless, to our knowledge, this is the first study that investigates LOH and MSI in samples of metachronous esophageal cancers developed in HL or breast cancer long-term survivors.

LOH and MSI frequencies were evaluated at chromosomal regions known to be frequently altered in sporadic esophageal cancer with particular attention to loci nearby genes involved in DNA double strand break (DSB) or in the oxidative damage repair pathways (i.e. *BRCA1*, *BRCA2*, *RAD1* and *MSRA*).

Results showed that the overall LOH frequency was similar among metachronous and sporadic esophageal tumors for the majority of the analyzed regions. However, the regions probed by D8S1130 (8p23.1), D13S260 (13q13.1), D17S1323 and D17S1327 (17q21.31) exhibited lower LOH frequency in metachronous tumors than in the sporadic ones with the D17S1327 reaching a statistical significance (p = 0.018). Altogether, these findings are quite intriguing since the microsatellite markers used to investigate these regions map near to *BRCA2* (352 Kb) and *BRCA1* (95 Kb and 232 Kb) genes (see Table 2), two key oncosuppressor genes involved in DSB repair pathway [36]. DSB repair is a typical repair mechanism of ionizing radiation-induced DNA damage, therefore we expected a major involvement of these regions in the therapy-related metachronous cancers [37]. On the contrary, the same regions resulted to be more stable in the latter than in the sporadic esophageal cancers.

Furthermore, when we considered separately, our data pointed out some differences between ESCC and EADC, suggesting that some chromosomal abnormalities are mainly related to tumor histotype rather than to therapy. Indeed, allelic losses at chromosomal regions 3p24.1, 5q11.2, 9p21.3 and 13q13.2, were more common in metachronous ESCC than in metachronous EADC, as observed herein, and previously reported, for sporadic ESCC and EADC [12].

These findings might suggest that alterations at the above mentioned regions are causal in the development of ESCC but incidental in the carcinogenic process of EADC.

High LOH frequency at the 5p12 chromosomal region, probed by the microsatellite marker D5S2106, was observed in metachronous EADC; in contrast, sporadic EADC and both forms of ESCC showed low LOH frequency at this locus. Interestingly, the *RAD1* gene, involved in DSB repair, maps near the region probed by the used microsatellite marker. Thus, it seems that no distinct molecular markers, able to discriminate between therapy-related and spontaneous ESCC, exist. On the contrary, the high LOH frequency at the 5p12 region found only in the metachronous EADC might suggest the presence in this region of a genetic determinant linked to the development of therapy-related EADC.

We also found that sporadic ESCC and EADC exhibited a higher fractional allelic loss (FAL) index than their respective therapy-related tumors, suggesting that transformation processes in the latter might require fewer events than in the sporadic counterpart. MSI frequency was very low in both groups at all analyzed regions. This finding is in agreement with previous studies on sporadic esophageal cancers indicating MSI as a rare event in both esophageal tumor histotypes [25, 27, 28]. This data also indicates that therapy-related metachronous esophageal cancers do not have the mutator phenotype that is usually considered as a paradigm for cell transformation after radiation exposure [38, 39].

In conclusion, no high-level peculiar genetic alterations were found in therapy-related esophageal carcinomas, suggesting that their transformation process retraces the one that occurs in the sporadic forms. We also found that therapy-related esophageal cancers reiterate the molecular differences between ESCC and EADC highlighting the existence of a histotype-specific signature.

## Supporting Information

S1 Table PCR conditions and size of related products. (DOC)

## References

[1] Dores GM, Metayer C, Curtis RE, Lynch CF, Clarke EA, et al (2002) Second malignant neoplasms among long-term survivors of Hodgkin′s disease: a population-based evaluation over 25 years. J Clin Oncol 20: 3484–3494. 10.1200/JCO.2002.09.038 12177110

[2] Ng AK, Travis LB (2008) Second primary cancers: an overview. Hematol Oncol Clin North Am 22: 271–289. 10.1016/j.hoc.2008.01.007 18395150

[3] Fekete F, Mosnier H, Belghiti J, Uribe M, Sauvanet A (1994) Esophageal cancer after mediastinal irradiation. Dysphagia 9: 289–291. 8005009

[4] Micke O, Schafer U, Glashorster M, Prott FJ, Willich N (1999) Radiation-induced esophageal carcinoma 30 years after mediastinal irradiation: case report and review of the literature. Jpn J Clin Oncol 29: 164–170. 10.1093/jjco/29.3.164 10225701

[5] Clarke M, Collins R, Darby S, Davies C, Elphinstone P, et al (2005) Effects of radiotherapy and of differences in the extent of surgery for early breast cancer on local recurrence and 15-year survival: an overview of the randomised trials. Lancet 366: 2087–2106. 10.1016/S0140-6736(05)67887-7 16360786

[6] Morton LM, Gilbert ES, Hall P, Andersson M, Joensuu H, et al (2012) Risk of treatment-related esophageal cancer among breast cancer survivors. Ann Oncol 23: 3081–3091. 10.1093/annonc/mds144 22745217 PMC3501231

[7] Stoner GD, Gupta A (2001) Etiology and chemoprevention of esophageal squamous cell carcinoma. Carcinogenesis 22: 1737–1746. 10.1093/carcin/22.11.1737 11698334

[8] Chen X, Yang CS (2001) Esophageal adenocarcinoma: a review and perspectives on the mechanism of carcinogenesis and chemoprevention. Carcinogenesis 22:1119–1129. 10.1093/carcin/22.8.1119 11470739

[9] Pennathur A, Gibson MK, Jobe BA Luketich JD (2013) Oesophageal carcinoma. Lancet 381:400–412. 10.1016/S0140-6736(12)60643-6 23374478

[10] Hu N, Roth MJ, Polymeropolous M, Tang ZZ, Emmert-Buck MR, et al (2000) Identification of novel regions of allelic loss from a genomewide scan of esophageal squamous-cell carcinoma in a high-risk Chinese population. Genes Chromosomes Cancer 27: 217–228. 10.1002/(SICI)1098-2264(200003)27:3<217::AID-GCC1>3.0.CO;2-A 10679910

[11] Dulak AM, Schumacher SE, van Lieshout J, Imamura Y, Fox C, et al (2012) Gastrointestinal adenocarcinomas of the esophagus, stomach, and colon exhibit distinct patterns of genome instability and oncogenesis. Cancer Res 72: 4383–4393. 10.1158/0008-5472.CAN-11-3893 22751462 PMC3432726

[12] Rumiato E, Pasello G, Montagna M, Scaini MC, De Salvo GL, et al (2011) DNA copy number profile discriminates between esophageal adenocarcinoma and squamous cell carcinoma and represents an independent prognostic parameter in esophageal adenocarcinoma. Cancer Lett 310: 84–93. 10.1016/j.canlet.2011.06.017 21757289

[13] Peralta RC, Casson AG, Wang RN, Keshavjee S, Redston M, et al (1998) Distinct regions of frequent loss of heterozygosity of chromosome 5p and 5q in human esophageal cancer. Int J Cancer 78: 600–605. 10.1002/(SICI)1097-0215(19981123)78:5<600::AID-IJC12>3.0.CO;2-1 9808529

[14] Bandla S, Pennathur A, Luketich JD, Beer DG, Lin L, et al (2012) Comparative genomics of esophageal adenocarcinoma and squamous cell carcinoma. Ann Thorac Surg 93: 1101–1116. 10.1016/j.athoracsur.2012.01.064 22450065 PMC3401935

[15] Chattopadhyay I, Singh A, Phukan R, Purkayastha J, Kataki A, et al (2010) Genome-wide analysis of chromosomal alterations in patients with esophageal squamous cell carcinoma exposed to tobacco and betel quid from high-risk area in India. Mutat Res 696: 130–138. 10.1016/j.mrgentox.2010.01.001 20083228

[16] Ko JM, Wong CP, Tang CM, Lau KW, Lung ML (2001) Frequent loss of heterozygosity on multiple chromosomes in Chinese esophageal squamous cell carcinomas. Cancer Lett, 170: 131–138. 10.1016/S0304-3835(01)00577-8 11463490

[17] Dolan K, Garde J, Gosney J, Sissons M, Wright T, et al (1998) Allelotype analysis of oesophageal adenocarcinoma: loss of heterozygosity occurs at multiple sites. Br J Cancer 78: 950–957. 10.1038/bjc.1998.607 9764589 PMC2063115

[18] Nancarrow DJ, Handoko HY, Smithers BM, Gotley DC, Drew PA, et al (2008) Genome-wide copy number analysis in esophageal adenocarcinoma using high-density single-nucleotide polymorphism arrays. Cancer Res 68: 4163–4172. 10.1158/0008-5472.CAN-07-6710 18519675

[19] Cai YC, So CK, Nie AY, Song Y, Yang GY, et al (2007) Characterization of genetic alteration patterns in human esophageal squamous cell carcinoma using selected microsatellite markers spanning multiple loci. Int J Oncol 30: 1059–1067. 17390007

[20] Tarmin L, Yin J, Zhou X, Suzuki H, Jiang HY, et al (1994) Frequent loss of heterozygosity on chromosome 9 in adenocarcinoma and squamous cell carcinoma of the esophagus. Cancer Res 54: 6094–6096. 7954453

[21] Lichun Y, Ching Tang CM, Wai Lau K, Lung ML (2004) Frequent loss of heterozygosity on chromosome 9 in Chinese esophageal squamous cell carcinomas. Cancer Lett 203: 71–77. 10.1016/j.canlet.2003.09.027 14670619

[22] Liu M, Zhang F, Liu S, Zhao W, Zhu J, et al (2011) Microsatellite analysis in multistage carcinogenesis of esophageal squamous cell carcinoma from chongqing in southern china. Int J Mol Sci 12: 7401–7409. 10.3390/ijms12117401 22174605 PMC3233411

[23] Mori T, Aoki T, Matsubara T, Iida F, Du X, et al (1994) Frequent loss of heterozygosity in the region including BRCA1 on chromosome 17q in squamous cell carcinomas of the esophagus. Cancer Res 54: 1638–1640. 7907942

[24] Montesano R, Hollstein M, Hainaut P (1996) Genetic alterations in esophageal cancer and their relevance to etiology and pathogenesis: a review. Int J Cancer 69: 225–235. 10.1002/(SICI)1097-0215(19960621)69:3<225::AID-IJC13>3.0.CO;2-6 8682592

[25] Araki K, Wang B, Miyashita K, Cui Q, Ohno S, et al (2004) Frequent loss of heterozygosity but rare microsatellite instability in oesophageal cancer in Japanese and Chinese patients. Oncology 67: 151–158. 10.1159/000081002 15539920

[26] Gleeson CM, Sloan JM, McGuigan JA, Ritchie AJ, Weber JL, et al (1996) Ubiquitous somatic alterations at microsatellite alleles occur infrequently in Barrett′s-associated esophageal adenocarcinoma. Cancer Res 56: 259–263. 8542577

[27] Evans SC, Gillis A, Geldenhuys L, Vaninetti NM, Malatjalian DA, et al (2004) Microsatellite instability in esophageal adenocarcinoma. Cancer Lett 212: 241–251. 10.1016/j.canlet.2004.03.011 15279904

[28] Muzeau F, Flejou JF, Belghiti J, Thomas G, Hamelin R (1997) Infrequent microsatellite instability in oesophageal cancers. Br J Cancer 75: 1336–1339. 10.1038/bjc.1997.226 9155055 PMC2228236

[29] De Benedetti VM, Travis LB, Welsh JA, van Leeuwen FE, Stovall M, et al (1996) p53 mutations in lung cancer following radiation therapy for Hodgkin′s disease. Cancer Epidemiol Biomarkers Prev 5: 93–98. 8850268

[30] Behrens C, Travis LB, Wistuba II, Davis S, Maitra A, et al (2000) Molecular changes in second primary lung and breast cancers after therapy for Hodgkin′s disease. Cancer Epidemiol Biomarkers Prev 9: 1027–1035. 11045784

[31] Ruol A, Castoro C, Portale G, Cavallin F, Sileni VC, et al (2009) Trends in management and prognosis for esophageal cancer surgery: twenty-five years of experience at a single institution. Arch Surg 144: 247–254. 10.1001/archsurg.2008.574 19289664

[32] Chai J, Jamal MM (2012) Esophageal malignancy: a growing concern. World J Gastroenterol 18: 6521–6526. 10.3748/wjg.v18.i45.6521 23236223 PMC3516225

[33] Brat DJ, James CD, Jedlicka AE, Connolly DC, Chang E, et al (1999) Molecular genetic alterations in radiation-induced astrocytomas. Am J Pathol 154: 1431–1438. 10.1016/S0002-9440(10)65397-7 10329596 PMC1866591

[34] Mertens F, Larramendy M, Gustavsson A, Gisselsson D, Rydholm A, et al (2000) Radiation-associated sarcomas are characterized by complex karyotypes with frequent rearrangements of chromosome arm 3p. Cancer Genet Cytogenet 116: 89–96. 10.1016/S0165-4608(99)00105-3 10640139

[35] Shoshan Y, Chernova O, Juen SS, Somerville RP, Israel Z, et al (2000) Radiation-induced meningioma: a distinct molecular genetic pattern? J Neuropathol Exp Neurol 59: 614–620. 10901233 10.1093/jnen/59.7.614

[36] Foulkes WD, Shuen AY (2013) In Brief: BRCA1 and BRCA2. J Pathol 230: 347–349. 10.1002/path.4205 23620175

[37] Little JB (2000) Radiation carcinogenesis. Carcinogenesis 21: 397–404. 10.1093/carcin/21.3.397 10688860

[38] Dubrova YE, Nesterov VN, Krouchinsky NG, Ostapenko VA, Neumann R, et al (1996) Human minisatellite mutation rate after the Chernobyl accident. Nature 380: 683–686. 10.1038/380683a0 8614461

[39] Morgan WF, Day JP, Kaplan MI, McGhee EM, Limoli CL (1996) Genomic instability induced by ionizing radiation. Radiat Res 146: 247–258. 10.2307/3579454 8752302

