# Correction: Genetic Features of Metachronous Esophageal Cancer Developed in Hodgkin’s Lymphoma or Breast Cancer Long-Term Survivors: An Exploratory Study

**DOI:** 10.1371/journal.pone.0121467

**Published:** 2015-03-23

**Authors:** The PLOS ONE Staff

There are formatting errors in Table 3 in the PDF version of the article. Please see the correct Table 3 here.

**Table 3:** LOH and MSI status detected in metachronous or sporadic ESCC and EADC patients.

SPORADIC										
Patient	D3S37273p24.1	D5S21065p12	D5S6235q11.2	D8S11308p23.1	D9S9429p21.3	D9S1719p21.3	D13S26013q13.1	D13S26713q13.2	D17S132317q21.31	D17S132717q21.31
**ESCC**										
01	●	○	●	●	○	●	●	Ø MSI	●	●
02	○	○	●	Ø	○	Ø	Ø	●	Ø	○
03	Ø MSI	Ø	○	Ø	○	Ø	●	●	○	●
04	●	Ø	●	Ø	●	Ø	●	Ø	●	Ø
05	●	Ø	Ø	Ø	●	○	Ø	○	Ø	Ø
06	●	○	Ø	○	○	● MSI	Ø	●	○	○
07	●	●	Ø	●	●	●	Ø	●	Ø	●
08	●	Ø	●	Ø	●	●	●	○	●	●
09	●	●	●	●	● MSI	Ø	●	●	●	Ø
10	Ø	●	●	●	○	●	●	●	Ø	○
11	●	○ MSI	○	○	○	○	○	○	○	○
12	○	○	Ø	●	●	●	●	Ø	○	Ø
13	○	●	●	●	○	●	Ø	●	○	○
14	○	○	○	○	○	○	●	Ø	○	○
15	●	Ø	Ø	○	●	●	●	●	○	●
**EADC**										
01	●	●	●	○ MSI	● MSI	○	○	●	Ø	Ø
02	○	○	Ø	●	○	Ø	Ø	●	Ø	Ø
03	○	○	Ø	●	○	○	Ø	●	Ø	○
04	●	Ø	○	●	○	○	○	●	Ø	●
05	Ø	●	Ø	●	○	●	Ø	●	Ø	●
06	● MSI	Ø MSI	○ MSI	Ø MSI	○	○ MSI	○	○ MSI	○ MSI	●
07	Ø	●	●	Ø	●	●	●	●	●	●
08	○	Ø	○	●	●	Ø	DEL	○	○	●
09	Ø	○	○	Ø	○	●	○	○	Ø	Ø
10	Ø	Ø	○	●	●	●	●	Ø	Ø	Ø
11	●	●	●	●	●	Ø	●	●	●	Ø
Pos/inf (%)	13/20(65)	8/17(47)	10/18(56)	13/18(72)	12/26(46)	12/19(63)	13/18(72)	15/21(71)	6/15(40)	10/17(59)

**METACHRONOUS**										
**Patient**	**D3S37273p24.1**	**D5S21065p12**	**D5S6235q11.2**	**D8S11308p23.1**	**D9S9429p21.3**	**D9S1719p21.3**	**D13S26013q13.1**	**D13S26713q13**	**D17S132317q21.31**	**D17S132717q21.31**
**ESCC**										
01	●	○	●	●	○	●	Ø	●	Ø	Ø
02	○	Ø	●	Ø MSI	Ø	●	○	●	●	Ø
03	●	●	Ø	Ø	●	○	Ø	●	○	○
04	●	○	Ø	○	○	Ø	Ø	●	○	○
05	○	Ø	●	○	○	Ø	○	Ø	○	○
06	Ø	●	●	Ø	○	●	●	●	○	●
07	●	NA	NA	Ø MSI	●	●	○	NA	NA	Ø
08	●	○	Ø	○	○	●	○	Ø	○	○
09	○	○	●	Ø	○	●	Ø	Ø	Ø	Ø
10	●	○	●	Ø	●	Ø	○	Ø	Ø	○
11	Ø	●	○	●	○	○	●	○	○	○
12	●	●	●	○	●	●	●	Ø	Ø	Ø MSI
13	Ø	●	●	●	●	●	●	●	○	Ø
14	●	○	Ø	○	●	●	Ø	○	Ø	Ø
**EADC**										
01	○	●	●	○	● MSI	○	●	Ø	Ø	Ø
02	○	Ø	○	○	○	Ø	Ø	○	Ø	○
03	●	●	○	Ø	○	●	Ø	●	Ø	○
04	Ø	Ø	Ø	●	○	●	○	○	●	○
05	Ø	●	○	●	●	○	●	●	Ø	Ø
06	Ø	●	Ø	○	○	○	○	Ø	○	Ø
Pos/inf (%)	9/14(64)	9/15(60)	9/13(69)	5/13(38)	8/19(42)	11/16(69)	6/13(46)	8/12(67)	2/10(20)	1/10(10)
**p-value**	**1**	**0.50**	**0.48**	**0.07**	**1**	**1**	**0.26**	**1**	**0.4**	**0.018**

(●) LOH positive; (○) LOH negative; (Ø) uninformative; MSI (positive to microsatellite instability); NA (not amplifiable); DEL: deletion. p-value was calculated using the two-tailed Fisher’s exact test.
