# Supplementary material for: Genetic Features of Metachronous Esophageal Cancer Developed in Hodgkin’s Lymphoma or Breast Cancer Long-Term Survivors: An Exploratory Study
Source: PLoS One. 2015 Jan 22;10(1):e0117070. doi: 10.1371/journal.pone.0117070 (PMC4303414; doi:10.1371/journal.pone.0117070)
Supplement: S1 Table — (DOC) [file pone.0117070.s001.doc]

|  |  |  |  |
| --- | --- | --- | --- |
|  | **Supplementary Table S1.** PCR conditions and size of related products. | | |
|  | **Microsatellite** | **PCR Conditions** | **PCR Product Size (bp)** |
|  |  | 38 cycles: 94°C 1 min, 54°C 1 min, 72°C 50 s, final extension: 72C° 45 min |  |
|  | D3S3727 |  | 115-135 bps |
|  |  |  |  |
|  |  | 35 cycles: 94°C 1 min, 60°C 1 min, 72°C 50 s, final extension: 72°C 45 min |  |
|  | D5S2106 |  | 195-201 bps |
|  |  |  |  |
|  | D5S623 |  | 143-159 bps |
|  |  |  |  |
|  |  | 38 cycles: 94°C 1 min , 58°C 1 min, 72°C 50 s, final extension: 72°C 15 min |  |
|  | D8S1130 |  | 145 bps |
|  |  |  |  |
|  | D9S171 |  | 158-177 bps |
|  |  |  |  |
|  |  | 35 cycles: 95°C 30 s, 50°C 40 s, 72°C 40 s, final extension: 72°C 20 s |  |
|  | D9S942 |  | 100 bps |
|  |  |  |  |
|  |  | 35 cycles: 94°C 1 min, 58°C 1 min, 72°C 1 min, final extension: 72°C 45 min |  |
|  | D13S260 |  | 158-173 bps |
|  |  |  |  |
|  | D13S267 |  | 148-162 bps |
|  |  |  |  |
|  |  | 32 cycles: 94°C 1 min, 58°C 1 min, 72°C 1 min, final extension: 72°C 45 min |  |
|  | D17S1323 |  | 155 bps |
|  |  |  |  |
|  | D17S1327 |  | 130 bps |
|  |  |  |  |
|  |  |  |  |
